# Co-morbid gambling disorder in a local drug and alcohol service: an audit to determine prevalence

**DOI:** 10.1192/bjo.2021.477

**Published:** 2021-06-18

**Authors:** John Barker, Ruta Rele, Charlotte Cartwright, Bethany Dinsdale-Young

**Affiliations:** 1 Sheffield Health and Social Care NHS Foundation Trust; 2 The University of Sheffield; 3 The University of Sheffield

## Abstract

**Aims:**

National surveys show that over 56% of adults in England gamble annually, and of those surveyed, 0.5% were problem gamblers, equating to 300,000 problem gamblers at any point. The prevalence of problem gambling in patients with a substance misuse disorder ranges from 20.5% to 55%.

The audit aims to improve the care of patients with comorbid substance misuse and gambling disorder by assessing the extent to which the service currently enquires about and records problem gambling in its patient cohort.

It is hypothesised that as no formal recording process is in place locally, this information will not be recorded systematically and in a way that is easily retrievable by the service.

The audit will allow the service to assess whether changes need to be made to the initial assessment pathways into treatment for substance-related disorders to adequately record this information so that further assessment and onward referral can take place.

**Method:**

All active patients (n = 2824) within the service had both their electronic initial assessments and their entire electronic notes screened for terms such as ‘betting’ and ‘gambling’ and this was recorded using an Excel spreadsheet. Prevalence rates across the teams (opiates, non-opiates and alcohol) were then calculated.

**Result:**

The results showed that 0% of patients had any entries in their initial screening noting any gambling activity. Further scrutiny of the records revealed that only 3.5% (n-99) had ever discussed gambling with a worker in any of the services.

**Conclusion:**

The majority (n = 52) of patients who had discussed gambling only had one positive search result, suggesting this was not followed-up in a systematic fashion. Recommendations are to revise the common assessment pro-forma to include a validated brief screening tool (lie/bet), where one positive answer triggers a further assessment with an appropriate clinician for consideration of referral to the local NHS gambling service.

